# Soybean Lecithin–Gallic Acid Complex Sensitizes Lung Cancer Cells to Radiation Through Ferroptosis Regulated by Nrf2/SLC7A11/GPX4 Pathway

**DOI:** 10.3390/nu17071262

**Published:** 2025-04-03

**Authors:** Xingyang Chen, Hongli Cui, Lijing Qin, Rongrong Liu, Fang Fang, Zhicheng Wang

**Affiliations:** 1 Department of Nutrition and Food Hygiene, School of Public Health, Jilin University, Changchun 130021, China; 2 NHC Key Laboratory of Radiobiology, School of Public Health, Jilin University, Changchun 130021, China

**Keywords:** soybean lecithin–gallic acid, non-small cell lung cancer, ionizing radiation, radiosensitivity

## Abstract

**Background**: Radioresistance remains a significant obstacle in lung cancer radiotherapy, necessitating novel strategies to enhance therapeutic efficacy. This study investigated the radiosensitizing potential of a soybean lecithin–gallic acid complex (SL–GAC) in non-small cell lung cancer (NSCLC) cells and explored its underlying ferroptosis-related mechanisms. SL–GAC was synthesized to improve the bioavailability of gallic acid (GA), a polyphenol with anticancer properties. **Methods**: NSCLC cell lines (A549 and H1299) and normal bronchial epithelial cells (BEAS-2B) were treated with SL–GAC, ionizing radiation (IR), or their combination. Through a series of in vitro experiments, including cell viability assays, scratch healing assays, flow cytometry, and Western blot analysis, we comprehensively evaluated the effects of SL-GAC on NSCLC cell proliferation, migration, oxidative stress, and ferroptosis induction. **Results**: SL–GAC combined with IR synergistically suppressed NSCLC cell proliferation and migration, exacerbated oxidative stress via elevated ROS and malondialdehyde levels, and induced mitochondrial dysfunction marked by reduced membrane potential and structural damage, whereas no significant ROS elevation was observed in BEAS-2B cells. Mechanistically, the combination triggered ferroptosis in NSCLC cells, evidenced by iron accumulation and downregulation of Nrf2, SLC7A11, and GPX4, alongside upregulated ACSL4. Ferrostatin-1 (Fer-1), a ferroptosis inhibitor, reversed these effects and restored radiosensitivity. **Conclusions**: Our findings demonstrate that SL–GAC enhances NSCLC radiosensitivity by promoting ferroptosis via the Nrf2/SLC7A11/GPX4 axis, highlighting its potential as a natural radiosensitizer for clinical translation.

## 1. Introduction

Globally, lung cancer ranks second among the leading causes of cancer mortality, exhibiting a dismal 5-year overall survival rate below 20% [1]. Non-small cell lung cancer (NSCLC) constitutes approximately 85% of all diagnosed lung malignancies [2]. Treatment strategies for NSCLC encompass a range of modalities, such as surgery, radiotherapy, chemotherapy, interventional radiology, and palliative care [3]. Radiotherapy, using ionizing radiation (IR) as the primary modality, constitutes a significant therapeutic approach in the management of lung cancer [4]. However, a substantial proportion of lung cancer patients undergo tumor recurrence and metastasis after radiotherapy, with approximately half occurring within the radiation field [5]. Radiosensitizers are chemical entities or agents specifically designed to augment the lethal effects of radiotherapy on tumor cells. They function by promoting accelerated DNA damage and indirectly triggering the production of free radicals, thereby effectively overcoming tumor resistance to radiotherapy [6]. Despite ongoing efforts to develop diverse radiosensitizers, currently available options remain inadequate for clinical needs [7]. Consequently, advancing the development of novel radiosensitizers with optimized therapeutic efficacy emerges as a critical priority in contemporary radiation oncology.

Recently, numerous studies have demonstrated that active compounds from Chinese herbs, such as curcumin [8,9], paclitaxel [10], and resveratrol [11], can enhance tumor radiosensitivity. Gallic acid (GA), a natural plant chemical, has been shown to have anti-tumor properties [12,13,14]. However, GA has low cell membrane permeability and stability, resulting in low absorption and utilization rates and suboptimal therapeutic effects [15,16]. We previously synthesized a complex drug, SL–GAC, by utilizing soy lecithin as a carrier to enhance the lipo-solubility of GA, thereby improving its pharmacological properties and biological utilization [17]. In this study, we explored the potential of SL–GAC as a radiosensitizer for NSCLC by employing a combination of SL–GAC and ionizing radiation (IR) to treat NSCLC cells (A549, H1299). Our findings reveal that SL–GAC not only suppresses the proliferation and migration of NSCLC cells but also augments their radiosensitivity through the induction of ferroptosis. Therefore, this study validates the cytotoxic effects of SL–GAC against NSCLC cells and underscores its promising potential as a radiosensitizer for NSCLC.

## 2. Materials and Methods

### 2.1. Cell Culture

The human non-small cell lung cancer cell lines, A549 (CCL-185) and H1299 (CRL-5803), were obtained from American Type Culture Collection (ATCC, Manassas, VA, USA). Normal human bronchial epithelial (BEAS-2B, GNHu27) cell lines were purchased from the Chinese Academy of Sciences (Shanghai, China). Cells were propagated in Dulbecco’s Modified Eagle Medium (DMEM, Gibco, Grand Island, NY, USA) media containing 10% Fetal bovine serum (FBS, Biological Industries, Kibbutz Beit Haemek, Israel) and penicillin–streptomycin (Gibco, Grand Island, NY, USA) at 37 °C under a humidified 5% CO_2_ atmosphere.

### 2.2. Preparation and Properties of SL–GAC

According to the method described previously, SL–GAC is a substance different from the monomer complex of GA and lecithin, which is obtained by using GA and lecithin at the optimal reaction temperature (50 °C) for 3 h [17].

### 2.3. X-Ray Irradiation

The cells were irradiated using the X-RAD 320i X biological irradiator (Pacific X-ray Imaging, San Diego, CA, USA). The irradiation conditions were as follows: voltage-180 kV, current-12.0 mA, target skin distance-70 cm, and dose rate-1.02 Gy/min.

### 2.4. Cell Viability Assay

Cell viability was assessed using the Cell Counting Kit-8 (CCK-8; Beyotime Biotechnology, Shanghai, China) according to the manufacturer’s protocol. Absorbance was measured at 450 nm using a microplate reader (Sartorius AG, Göttingen, Germany).

### 2.5. The Scratch Healing Assay

To assess cell migration capacity, a standardized scratch wound healing assay was performed [18,19]. Briefly, cancer cells were cultured in 6-well plates until reaching 90–95% confluency as a monolayer. Uniform mechanical wounds were created using sterile 200 μL pipette tips with ruler-guided vertical scratching to ensure consistent width. After three gentle PBS washes to remove dislodged cells, fresh medium containing SL–GAC was added. Cells were incubated under standard conditions (37 °C, 5% CO_2_) for 24 h, with fixed-position imaging at baseline (0 h) and endpoint using an inverted phase-contrast microscope (Olympus IX73, Shinjuku, Tokyo, Japan).

### 2.6. Cell Cycle and Apoptosis Analysis by Flow Cytometry (FCM)

Cells pretreated with SL–GAC for 3 h were irradiated and cultured for 24 h before simultaneous cell cycle and apoptosis evaluation. Cells underwent dual washing with ice-cold PBS at 4 °C followed by fixation in 70% ethanol at −20 °C for 2 h. Nuclear DNA was stained using Beyotime C1052-2 PI solution at 50 μg/mL with 37 °C dark incubation for 30 min, and cell cycle phases were quantified via FCM. For apoptosis detection, cells pre-cultured in 6-well plates at 1 × 10^5^ density for 48 h were treated with SL–GAC for 24 h, washed twice with cold PBS, and dual-stained using Beyotime C1407 Annexin V-FITC/PI reagents. Annexin V binding was performed in calcium-enriched buffer at pH 7.4 with parallel PI counterstaining at 5 μg/mL, followed by immediate flow cytometric analysis [20].

### 2.7. Determination of ROS, Malondialdehyde (MDA) and Intracellular Iron Levels

In accordance with the manufacturer’s instructions, the intracellular ROS was measured using a peroxide-sensitive fluorescent probe DCFH-DA (Beyotime Biotechnology, Shanghai, China). Briefly, cells were incubated with 10 μM of DCFH-DA at 37 °C for 1 h, and the fluorescence intensity was calculated with flow cytometry. Based on the manufacturer’s instructions, the contents of MDA were measured by relevant reagent kits (Nanjing Jiancheng Bioengineering Institute, Nanjing, Jiangsu, China). A549 and H1299 cells were harvested and ultrasonicated. Next, according to the protein concentration of cells, MDA contents were evaluated and normalized. The contents of iron in cells were determined according to the manufacturer’s instructions (Beijing Applygen Technologies, Beijing, China).

### 2.8. Measurement of Mitochondrial Membrane Potential (MMP) Levels

The method for measuring mitochondrial membrane potential was performed following the previously reported protocol [21]. After treatment, the cells were washed twice with cold PBS and incubated in the dark at 37 °C with 5 μg/mL Rh123 (Sigma-Aldrich, St. Louis, MO, USA) for 30 min. The fluorescence of Rh123 was then detected using a flow cytometer.

### 2.9. Western Blot Analysis

Total protein lysates were prepared using radioimmunoprecipitation assay (RIPA) lysis buffer (Beyotime Biotechnology, Shanghai, China) containing a protease inhibitor cocktail (Beyotime Biotechnology, Shanghai, China). Proteins were separated on sodium dodecyl sulfate–polyacrylamide gel electrophoresis (SDS-PAGE) gels and transferred onto polyvinylidene fluoride (PVDF) membranes (Millipore Corporation, Billerica, MA, USA). After blocking with 5% nonfat milk, membranes were incubated with primary antibodies at 4 °C overnight. Primary antibodies against glutathione peroxidase 4 (GPX4), solute carrier family 7 member 11 (SLC7A11), acyl-CoA synthetase long-chain family member-4 (ACSL4), and glyceraldehyde-3-phosphate dehydrogenase (GAPDH) were purchased from Bioworld Technology, Inc. (Bloomington, MN, USA). Subsequently, the membranes were incubated with horseradish peroxidase (HRP)-linked goat anti-rabbit/mouse IgG secondary antibody (Bioworld, Bloomington, MN, USA) for 2 h at room temperature. After three washes with Tris-buffered saline with Tween 20 (TBST), the membranes were visualized by chemiluminescence using an enhanced chemiluminescence (ECL) detection system.

### 2.10. Statistical Analysis

Data are presented as mean ± standard deviation (SD). Intergroup differences were analyzed using two-tailed Student’s *t*-test (two groups) or one-way ANOVA with post-hoc tests (≥3 groups). Statistical analyses were performed with SPSS 24.0 (IBM, Armonk, NY, USA) and GraphPad Prism 10.0 (GraphPad Software, San Diego, CA, USA). A *p*-value < 0.05 was considered statistically significant.

## 3. Results

### 3.1. SL–GAC Suppresses NSCLC Cell Proliferation and Synergizes with IR to Promote ROS Generation

**Figure 1:**
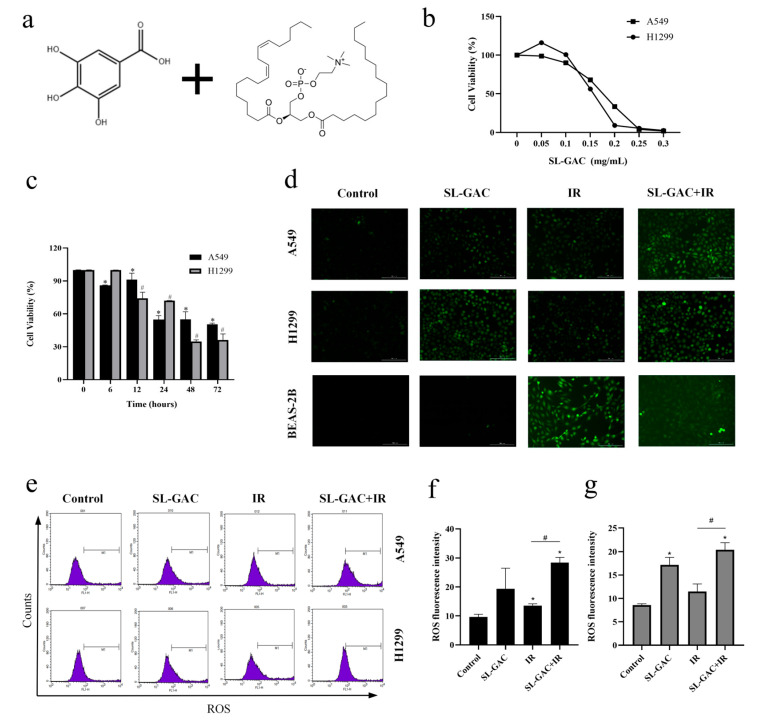
SL–GAC Inhibits NSCLC Proliferation and Enhances Tumor-Specific ROS with IR. (**a**) Preparation and properties of SL–GAC. (**b**,**c**) SL–GAC inhibited the proliferation of A549 and H1299 cells in a dose (**b**) and time (**c**) dependent manner. (**d**) SL–GAC and IR treatment upregulated ROS in A549 and h1299 cells, but SL–GAC alone did not affect ROS in Base2-B cells. (**e**–**g**) SL–GAC alone or combined with IR significantly elevates ROS levels in A549 (**f**) and H1299 (**g**) cells, as measured by DCFH-DA probe. Data represented as means ± SD, * p < 0.05, versus control group, # *p* < 0.01 versus SL–GAC+IR group. Data are representative from three parallel experiments.

To estimate the inhibitory effect of SL–GAC (Figure 1a) on NSCLC cells, A549 and H1299 cells were treated with different concentrations of SL–GAC. The CCK-8 assay was used to evaluate the effect of SL–GAC on cell proliferation. The results showed that SL–GAC inhibited the proliferation of A549 and H1299 cells in a dose- and time-dependent manner (Figure 1b,c). After 24 h, the half-maximal inhibitory concentration (IC50) values of SL–GAC for A549 and H1299 cells were determined to be 0.16 mg/mL and 0.14 mg/mL, respectively.

The existing studies indicate that the killing effect of IR on tumor cells is related to oxidative damage [22]. We detected the levels of ROS in cells to evaluate the effect of SL–GAC and IR treatment on oxidative stress in A549 and H1299 cells (Figure 1d). The DCFH-DA probe was used to analyze the level of ROS in cells. The results showed that, compared with the control group, SL–GAC treatment upregulated the level of ROS in cells (Figure 1e–g). The increase in ROS in the group treated with SL–GAC and IR was significantly greater than that in the SL–GAC or IR group (*p* < 0.05). However, SL–GAC did not induce a notable increase in ROS levels in BEAS-2B cells.

### 3.2. SL–GAC Enhances Radiosensitivity of A549 and H1299 Cells

**Figure 2:**
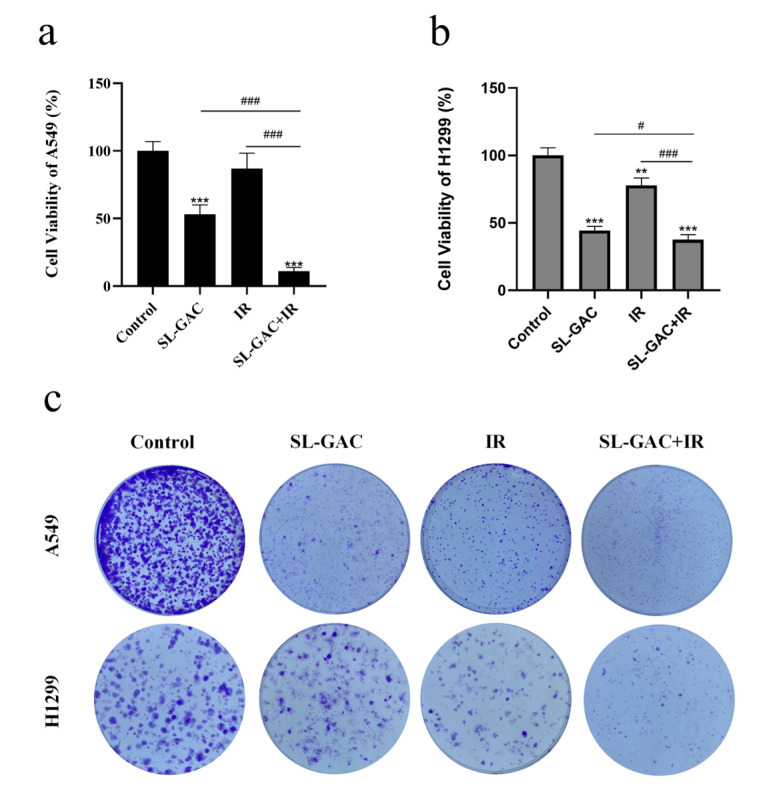
SL–GAC enhances the inhibitory effect of IR on the proliferation of A549 and H1299 cells. (**a**,**b**) SL–GAC Enhances Antiproliferation in NSCLC Cells with IR.0. (**c**) Combined SL–GAC and IR treatment abolishes colony formation in NSCLC cells. Data represented as means ± SD, ** *p* < 0.01, *** *p* < 0.001, versus control group; # *p*< 0.05, ### *p*< 0.001 versus SL–GAC+IR groups. Data are representative from three parallel experiments.

Then we investigated the effect of SL–GAC on the radiosensitivity of NSCLC cells. The CCK-8 assay was used to detect the effect of SL–GAC combined with ionizing radiation (IR) treatment on the proliferation of A549 and H1299 cells. The results showed that combined treatment inhibited the proliferation of A549 and H1299 cells (Figure 2a,b), and the inhibitory effect on proliferation was greater in the combined treatment group than in the SL–GAC or IR groups (*p* < 0.05). We further used a colony formation assay to detect the inhibitory effect on the proliferation of the combined treatment, and the results were consistent with those of the CCK-8 assay (Figure 2c). The combination of both treatment factors resulted in an almost complete loss of colony-forming ability in both lung cancer cells.

### 3.3. SL–GAC Enhanced IR-Mediated Mitochondrial Damage and Apoptosis in A549 and H1299 Cells

**Figure 3:**
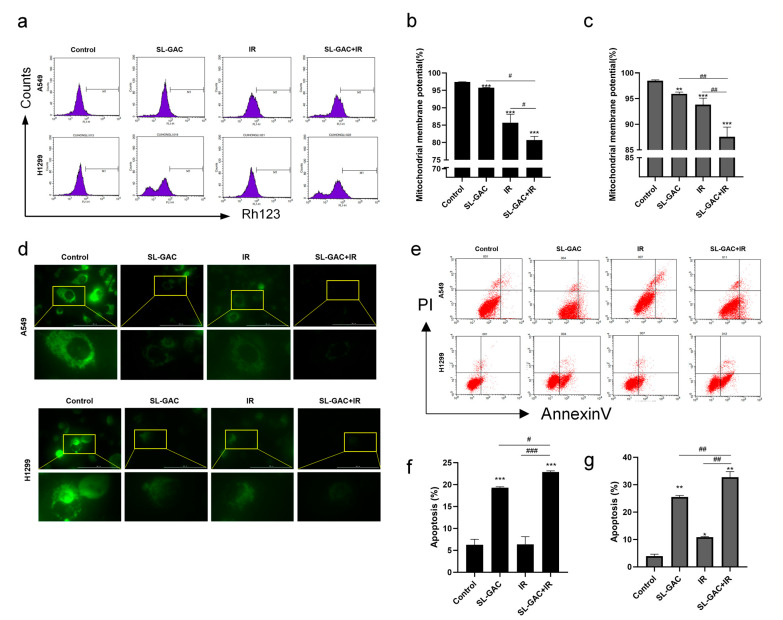
SL–GAC enhanced IR-mediated mitochondrial damage and apoptosis in A549 and H1299 cells. (**a**–**c**) The impact of SL–GAC and IR treatment on MMP level. (**d**) The impact of SL–GAC and IR treatment on mitochondrial integrity in A549 and H1299 cells. (**e**–**g**) Treatment with SL–GAC and IR promotes apoptosis in A549 and H1299 cells. Data represented as means ± SD, * *p* < 0.05, ** *p* < 0.01, *** *p* < 0.001 versus control group; # *p* < 0.05, ## *p* < 0.01, ### *p* < 0.001 versus SL–GAC+IR groups. Data are representative from three parallel experiments.

Flow cytometry was used to detect the changes of MMP in lung cancer cells after SL–GAC and IR treatment. The results showed that A549 and H1299 cells treated with SL–GAC had damaged mitochondria and decreased mitochondrial membrane potential (Figure 3a–c). This phenomenon was more significant in the group treated with SL–GAC and IR (*p* < 0.01). The Mito Tracker green probe was used to observe the effect of SL–GAC and IR treatment on the mitochondrial integrity of A549 and H1299 cells. As shown in Figure 3d, when treated with SL–GAC and IR, the fluorescence intensity of mitochondria decreased, and it was distributed less in the cytoplasm and appeared to be broken, indicating that the mitochondria of cells were severely damaged. To verify SL–GAC-mediated apoptosis in H1299 cells, Annexin V-FITC/PI dual staining followed by FCM analysis was performed. Both A549 and H1299 cells exhibited significant apoptosis after SL–GAC treatment, with a synergistic enhancement observed in the SL–GAC + IR combination group.

### 3.4. The Effect of SL–GAC in Combination with IR on the Cell Cycle and Migration Ability of A549 and H1299 Cells

**Figure 4:**
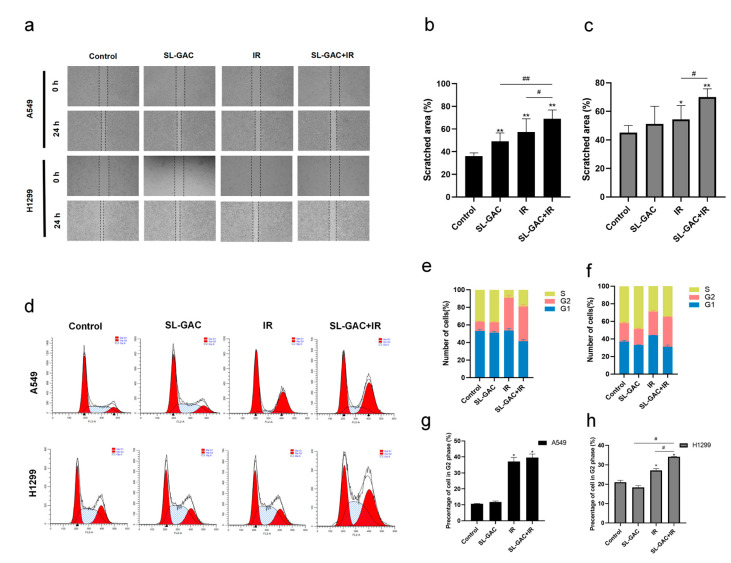
The effect of SL–GAC in combination with IR on the cell cycle and migration ability of A549 and H1299 cells. (**a**–**c**) The results of the scratch healing assay showed that combined treatment with SL–GAC and IR significantly inhibited cell migration. (**d**–**h**) SL–GAC and IR treatment caused a significant increase in the proportion of A549 and H1299 cells in G2 phase. Data represented as means ± SD, * *p* < 0.05, ** *p* < 0.01, versus control group; # *p* < 0.05, ## *p* < 0.01 versus SL–GAC+IR groups. Data are representative from three parallel experiments.

The scratch healing assay was used to detect the effect of SL–GAC and ionizing radiation combined treatment on the migration ability of A549 and H1299 cells. The results showed that the scratch in the control group healed significantly after 24 h, and the use of SL–GAC or ionizing radiation alone had a slight inhibitory effect on A549 and H1299 cells migration (Figure 4a,b). The cell cycle of A549 and H1299 cells after treatment was detected by flow cytometry. SL–GAC and IR treatment significantly increased the proportion of G2 phase (Figure 4d–h).

### 3.5. The Combination of SL–GAC and IR Treatments Induced Ferroptosis in A549 and H1299 Cells

**Figure 5:**
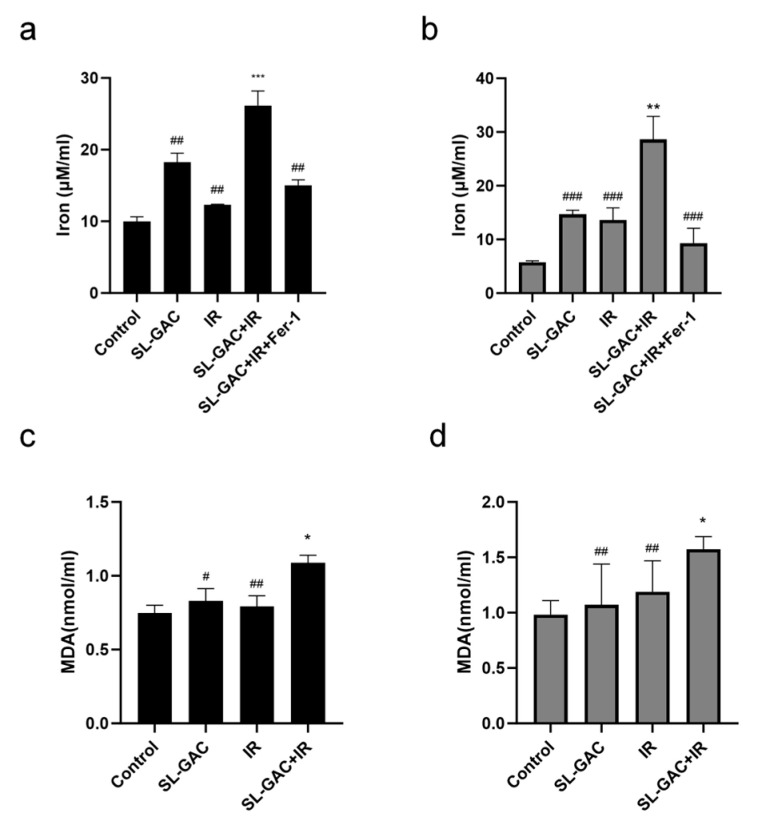
The combination of SL–GAC and IR treatments induced ferroptosis in A549 and H1299 cells. (**a**,**b**) The intracellular iron content of A549 (**a**) and H1299 (**b**) cells after treatment with SL–GAC and IR was significantly increased compared with that in the control group. (**c**,**d**) The level of MDA in the combined treatment group increased, and the combined effect was greater than IR treatment group in A549 (**a**) and H1299 (**b**) cells. Data are reported as mean ± SD. * *p* < 0.05, ** *p* < 0.01, *** *p* < 0.001 versus control group; # *p* < 0.05, ## *p* < 0.01, ### *p* < 0.001 versus SL–GAC+IR groups. Data are representative from three parallel experiments.

The intracellular iron colorimetric assay was used to detect changes in the intracellular iron content of A549 and H1299 cells after treatment with SL–GAC and IR. Notably, intracellular iron quantification demonstrated that while individual treatments (SL–GAC or IR) elevated cellular iron content versus untreated controls (*p* < 0.01), the combination regimen produced additive iron accumulation, showing statistically significant increases over both single-agent treatments (Figure 5a,b, *p* < 0.001). Cellular oxidative stress and oxidative damage are important mechanisms of ferroptosis in cells. MDA is commonly used to evaluate the degree of oxidative damage in cells. The level of MDA in the combined treatment group increased, and the combined effect was greater than the IR treatment group in A549 and H1299 cells (Figure 5c,d, *p* < 0.01). These collective findings demonstrate that the synergistic combination of SL–GAC and IR exacerbates ferroptosis in NSCLC cells through amplified iron accumulation and oxidative lipid damage.

### 3.6. Fer-1 Rescued Ferroptosis Induced by SL–GAC Treatment and the Resulting Radio Sensitization in A549 and H1299 Cells

**Figure 6:**
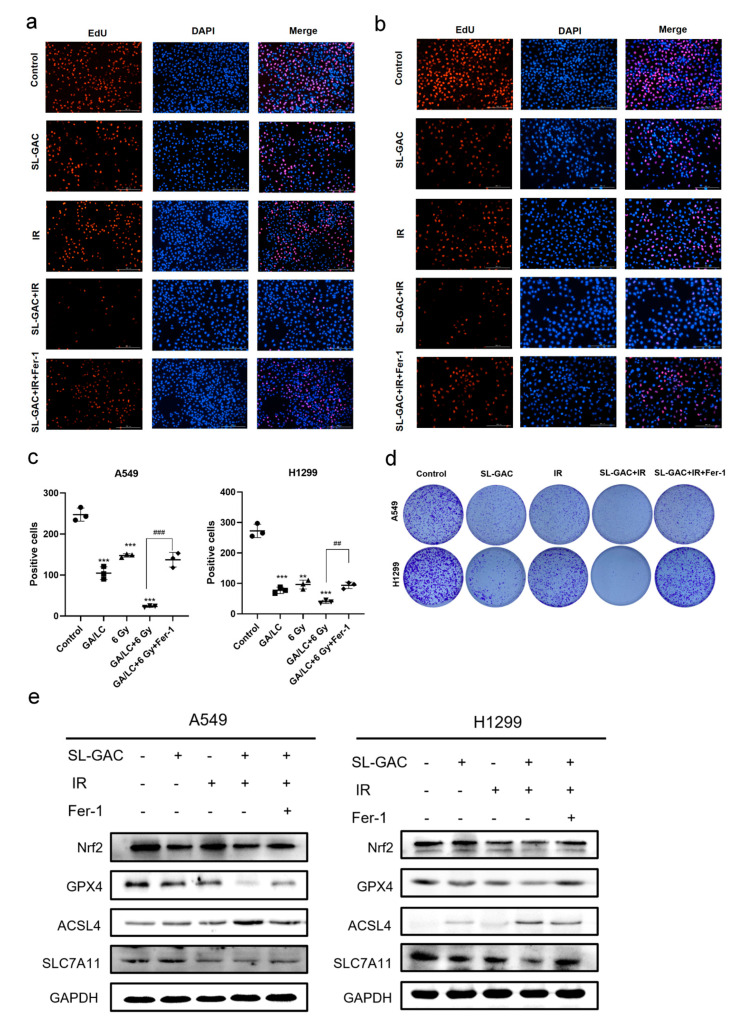
Fer-1 rescued ferroptosis induced by SL–GAC treatment and the resulting radio sensitization in A549 and H1299 cells. (**a**–**c**) Fer-1 rescues the SL–GAC-induced proliferation inhibition in A549 and H1299 cells. (**d**) Fer-1 rescued radio sensitization caused by SL–GAC in A549 and H1299 cells in clone formation experiments. (**e**) Fer-1 modulated the expression levels of iron death-related proteins in A549 and H1299 cells treated with SL–GAC and IR groups. ** *p* < 0.01, *** *p* < 0.001 versus control group; ## *p* < 0.01, ### *p* < 0.001 versus SL–GAC+IR groups. Data are representative from three parallel experiments.

We further explored whether the increase in radiotherapy sensitivity of A549 and H1299 cells by SL–GAC is related to ferroptosis. The EdU proliferation assay was used to detect cell proliferation. Proliferating cells were stained red under the fluorescence microscope. Compared with the control group, the red fluorescence intensity and number in both non-small cell lung cancer cells gradually decreased after 24 h of treatment with SL–GAC and IR, and the combined effect of SL–GAC and IR almost made the red fluorescence disappear (Figure 6a). Compared with the combined treatment group, the red fluorescence intensity and number of cells in the ferroptosis inhibitor Fer-1 pretreatment group were significantly restored. The results were quantitatively analyzed as shown in Figure 6c. Fer-1 can rescue the proliferation inhibitory effect of SL–GAC and radiation on lung cancer cells (*p* < 0.01). This indicates that ferroptosis is involved in the combined inhibition of lung cancer cell proliferation by SL–GAC and IR. The clone formation experiment results showed that when Fer-1 was used to pretreat both non-small cell lung cancer cells, compared with SL–GAC and IR alone or in combination, the clone formation ability of cells was significantly restored (Figure 6d). Fer-1 can rescue the inhibition of cell proliferation ability induced by SL–GAC and irradiation combined treatment. The above results indicate that the radiosensitivity of A549 cells by SL–GAC is related to ferroptosis. The WB experiment further verified the role of ferroptosis in SL–GAC increasing A549 and H1299 cells’ radiosensitivity. SL–GAC or IR irradiation alone can reduce the expression of Nrf2, GPX4, and SLC7A11 related to ferroptosis, while increasing ACSL4 expression (Figure 6e). The combined action of both can further reduce Nrf2 and GPX4 expression while increasing ACSL4 expression. Pretreatment with Fer-1 led to an upregulation of Nrf2, GPX4, and SLC7A11 expression, while ACSL4 expression decreased in comparison to the combined treatment group. This suggests that Fer-1 effectively rescues ferroptosis induced by SL–GAC and irradiation.

## 4. Discussion

Regulated cell death plays a pivotal role in preventing and treating diseases characterized by excessive cellular proliferation, such as cancer [23]. Among the various forms of cell demise, ferroptosis stands out as a recently identified type. Ferroptosis is a non-apoptotic form of cell death that occurs when imbalanced (ROS) attack cell membranes and cause peroxidation of polyunsaturated fatty acids [24,25,26]. Recently, research has demonstrated that IR utilized in radiotherapy has the capability to induce ferroptosis in tumor cells [4,27,28]. Certain ferroptosis inducers, such as Erastin and RSL3, have been widely studied as radiotherapy sensitizers that enhance the cytotoxic effect of IR on tumors [29]. Prior research has demonstrated that GA effectively suppresses tumor growth by restraining cell proliferation, inducing apoptosis, and activating autophagy, all while maintaining low toxicity and minimal side effects [13,19,20,30]. However, recent research has discovered that GA can also trigger ferroptosis in small cell lung cancer and neuroblastoma cells [31,32]. In our study, we investigated the inhibitory effects of SL–GAC on NSCLC cell lines (A549 and H1299) proliferation and its influence on radiosensitivity. CCK-8 and colony formation assays revealed that SL–GAC effectively suppressed A549 and H1299 cells proliferation. Moreover, the combined treatment exhibited greater efficacy. Cell migration represents a vital physiological process in normal cellular function, yet becomes pathologically distorted in malignancies through dysregulated proliferative and invasive behaviors. This metastatic progression is driven by coordinated molecular events including cytoskeletal reorganization mediated by actomyosin networks, dynamic modulation of focal adhesion complexes, and enzymatic extracellular matrix remodeling via matrix metalloproteinases in NSCLC. The epithelial–mesenchymal transition emerges as a pivotal regulatory mechanism, molecularly defined by transcriptional repression of the cell adhesion molecule E-cadherin coupled with concomitant induction of mesenchymal markers *N*-cadherin and vimentin, thereby conferring enhanced cellular motility and invasive capacity characteristic of aggressive tumor phenotypes [18,19]. Notably, the cell scratch assay showed that SL–GAC combined with IR significantly inhibited migration of A549 and H1299 cells compared to either treatment alone. ROS is one of the by-products of normal cell oxidative processes and an important mediator of intracellular signaling [33,34]. Therefore, we studied the possible role of ROS in SL–GAC and IR-mediated A549 and H1299 cell death. We found that after exposure to SL–GAC for 24 h, intracellular ROS increased in A549 and H1299 cells. Additionally, ionizing radiation has been reported to kill cancer cells through the ROS-mediated mitochondrial apoptotic pathway [35]. Mitochondrial membrane permeability is sensitive to oxidative stress, and excessive ROS can increase mitochondrial membrane permeability, leading to mitochondrial swelling and rupture, MMP depolarization, and the release of apoptosis-inducing proteins [34]. Our study found that SL–GAC and IR treatment disrupted mitochondrial integrity and significantly decreased the MMP level in A549 and H1299 cells. Oxidative stress is generally considered a major factor in many diseases, such as cancer, diabetes, asthma, and Parkinson’s disease [22,36]. MDA is a commonly used and widely accepted biomarker of oxidative stress [37]. We measured MDA levels in A549 and H1299 cells to determine the oxidative stress response SL–GAC and IR induced in lung cancer cells. The results showed that both SL–GAC and IR increased MDA levels in lung cancer cells, and the combination of the two factors significantly increased MDA levels. These results suggest that SL–GAC can increase the oxidative stress response of NSCLC cells induced by IR.

Radiotherapy is one of the most widely used cancer therapies, and many studies have demonstrated that ferroptosis can also be induced by radiotherapy [4,28,29,38]. IR-induced ROS can strip electrons from PUFA, leading to the formation of unstable fatty acid radicals (PUFA•). These radicals promptly react with oxygen molecules, giving rise to lipid peroxyl radicals (PUFA-OO•). Subsequently, these peroxyl radicals engage in reactions with other molecules, releasing H• via the Fenton reaction. Ultimately, this process culminates in the production of lipid hydroperoxides (PUFA-OOH), thereby initiating ferroptosis [39,40]. Our experimental findings demonstrate that the combined application of SL–GAC and IR effectively induces and potentiates oxidative stress in NSCLC cells, subsequently augmenting their radiosensitivity.

We therefore explored the potential link between SL–GAC-induced radiosensitivity and ferroptosis. The results of the iron quantification assay showed that the intracellular iron content significantly increased in A549 and H1299 cells after treatment with SL–GAC and IR, and this phenomenon could be rescued by iron-dependent cell death inhibitor Fer-1. The intracellular iron levels increase significantly during ferroptosis, which is an important marker of iron-dependent cell death [41]. The EdU proliferation assay showed that pretreatment with Fer-1 could rescue SL–GAC-induced A549 and H1299 cell death. ACSL4 is a key protein in the process of iron-dependent cell death. Studies have shown that IR can induce the expression of ACSL4 to increase the biosynthesis of PUFA-PL [39]. PUFA-PL and IR-induced ROS together drive the peroxidation of PUFA-PL to induce iron-dependent cell death, which is consistent with our WB results. IR also induces an adaptive response involving SLC7A11 or GPX4 to inhibit IR-induced iron-dependent cell death and promote the survival of cancer cells during radiotherapy [42,43,44]. The results of this study also showed that SL–GAC and IR can inhibit the expression of SLC7A11 or GPX4, and the combined effect of these two treatment factors further reduces the expression of SLC7A11 and GPX4, which can be rescued by the iron-dependent cell death inhibitor Fer-1. This further confirms that the radiation sensitizing effect of SL–GAC on A549 and H1299 cells is mediated through the iron-dependent cell death pathway. Nrf2 is a key transcription factor responsible for maintaining cellular metabolism, redox balance, and protein homeostasis, particularly during oxidative stress. Nrf2 regulates the expression of glutathione synthetase to prevent lipid peroxidation, making it a critical antioxidant. Inhibition of Nrf2 effectively enhances the susceptibility of cancer cells to ferroptosis [45,46]. The WB results showed that SL–GAC and IR can inhibit the expression of Nrf2, and Fer-1 can weaken the combined effect of SL–GAC and IR. This suggests that the Nrf2/SLC7A11/GPX4 pathway plays a crucial role in mediating the radiosensitizing effect of SL–GAC through ferroptosis. By targeting this pathway, SL–GAC holds promise as a potential radiosensitizer for enhancing the therapeutic efficacy of radiotherapy in NSCLC.

Intriguingly, while H1299 (p53-null) cells exhibited greater sensitivity to IR-mediated proliferative suppression compared to A549 (p53-wild-type) counterparts, they paradoxically demonstrated superior clonogenic survival capacity alongside more pronounced downregulation of the Nrf2/SLC7A11/GPX4 signaling axis. The observed differential responses between H1299 (p53-null) and A549 (p53-wild-type) cells may arise from p53-mediated compensatory mechanisms in redox regulation. In H1299 cells, constitutive activation of the Nrf2/SLC7A11/GPX4 axis due to p53 deficiency creates heightened dependency on this pathway for antioxidant defense, rendering them acutely sensitive to IR-induced pathway suppression, which triggers catastrophic lipid peroxidation and pronounced proliferation inhibition [47]. Paradoxically, their stronger pathway downregulation reflects baseline hyperactivation rather than therapeutic resistance, as complete suppression depletes their primary survival mechanism. Conversely, A549 cells leverage p53-dependent activation of alternative antioxidants (e.g., catalase, SOD2) to partially buffer against ferroptosis, allowing residual clonogenic survival despite milder pathway inhibition [48,49]. This dichotomy highlights how p53 status dictates therapeutic vulnerability: H1299’s “all-or-none” reliance on the targeted pathway amplifies acute cytotoxicity but limits recovery in clonogenic assays, while A549’s diversified redox network enables partial resistance through compensatory adaptations.

## 5. Conclusions

In summary, this study provides evidence that SL–GAC can enhance the radiosensitivity of NSCLC cells by inducing ferroptosis, and the Nrf2/SLC7A11/GPX4 pathway has important regulation roles, indicating its potential as a radiotherapy sensitizer for NSCLC.

## Data Availability

The original contributions presented in the study are included in the article, further inquiries can be directed to the corresponding author.
